# Lupeol, a Dietary Triterpene, Enhances Wound Healing in Streptozotocin-Induced Hyperglycemic Rats with Modulatory Effects on Inflammation, Oxidative Stress, and Angiogenesis

**DOI:** 10.1155/2019/3182627

**Published:** 2019-05-09

**Authors:** Fernando Pereira Beserra, Ana Júlia Vieira, Lucas Fernando Sérgio Gushiken, Eduardo Oliveira de Souza, Maria Fernanda Hussni, Carlos Alberto Hussni, Rafael Henrique Nóbrega, Emanuel Ricardo Monteiro Martinez, Christopher John Jackson, Gabriela Lemos de Azevedo Maia, Ariane Leite Rozza, Cláudia Helena Pellizzon

**Affiliations:** ^1^Department of Morphology, Institute of Biosciences, São Paulo State University (UNESP), Botucatu, São Paulo, Brazil; ^2^Department of Surgery and Veterinary Anesthesiology, School of Veterinary Medicine and Animal Science, São Paulo State University (UNESP), Botucatu, São Paulo, Brazil; ^3^Kolling Institute of Medical Research, The University of Sydney at Royal North Shore Hospital, Sydney, Australia; ^4^Department of Pharmacy, Federal University of São Francisco Valley (UNIVASF), Petrolina, Pernambuco, Brazil

## Abstract

Impaired wound healing is a debilitating complication of diabetes that leads to significant morbidity, particularly foot ulcers. Natural products have shown to be effective in treating skin wounds. Lupeol is known to stimulate angiogenesis, fibroblast proliferation, and expressions of cytokines and growth factors involved in wound healing. The study is performed to evaluate the wound healing activity of lupeol in streptozotocin-induced hyperglycemic rats by macroscopical, histological, immunohistochemical, immunoenzymatic, and molecular methods. Percentage of wound closure and contraction was increased in the lupeol-treated group when compared to the Lanette group. Histopathological observation revealed decreased inflammatory cell infiltration and increased proliferation of fibroblasts, vascularization, and deposition of collagen fibers after lupeol treatment. Immunohistochemical analyses showed decreased intensity of NF-*κ*B and increased intensity of FGF-2, TGF-*β*1, and collagen III. ELISA results revealed downregulated IL-6 levels and upregulated IL-10 levels in response to lupeol. The mRNA expression levels of *Hif-1α*, *Sod-2*, and *Ho-1* were significantly increased in response to lupeol as compared to Lanette whereas *Nf-κb* and *Vegf-A* levels were decreased in relation to insulin and lupeol treatment. These findings indicate that lupeol possesses wound healing potential in hyperglycemic conditions and may be useful as a treatment for chronic wounds in diabetic patients.

## 1. Introduction

Diabetes mellitus (DM) is a chronic metabolic disease, which is characterized by elevated levels of blood glucose leading, over time, to heart, blood vessel, eye, kidney, and nerve damage and failure to repair damage in skin wounds [1]. According to the World Health Organization (WHO), this disease affects 171 million people worldwide, and this number may be projected to reach approximately 366 million by 2030 [2]. The most common complication in patients with diabetes is an altered skin wound healing process, leading to complications such as Diabetic Foot Ulcers (DFUs), which has caused major worldwide morbidity due to various clinical and socioeconomic issues [3]. Several research studies have demonstrated that wound healing is delayed by hyperglycemia during DM [4–6].

In patients with diabetes, the mechanism of delayed wound healing has multifactorial causes, including a prolonged inflammatory stage and postponed proliferation and remodelling stages [7]. It has been reported that nuclear factor kappa B (NF-*κ*B) regulates the gene expression of several cytokines such as interleukin-1beta (IL-1*β*), interleukin-6 (IL-6), tumor necrosis factor-alpha (TNF-*α*), and interleukin-10 (IL-10); inducible nitric oxide synthase (iNOS); chemotactic and matrix proteins; immunological responses; and cell proliferation [8, 9]. The persistence of the inflammatory reaction is associated with oxidative stress, which is one of the most common complications for the delayed wound healing in diabetics [10]. The increased production of free radicals and decreased antioxidant activities of enzymes, such as superoxide dismutase (SOD), glutathione peroxidase (GPx), heme oxygenase-1, (HO-1) and heme oxygenase-2 (HO-2), may aggravate the situation leading to a delay in diabetic wound healing. Growth factors such as epidermal growth factor (EGF), fibroblast growth factor (FGF), transforming growth factor-beta1 (TGF-*β*1), and vascular endothelial growth factor (VEGF) and several molecules including hypoxia-inducible factor-1alpha (HIF-1*α*) are involved in the healing process by stimulating and activating cell proliferation via activation of various reactions, such as angiogenesis, reepithelialization, and differentiation and production of the extracellular matrix [11, 12]. In the present study, we investigated the role of the mediators involved in the healing process in hyperglycemic rat wounds.

There have been several attempts to accelerate the wound healing in diabetics, but so far, the therapeutic options available in the market are still limited due to the high cost, prolonged duration of treatment, and side effects. Therapeutics involving new molecules isolated from natural, safer products is an attractive alternative [3].

Several studies have suggested that plant-derived secondary metabolites are capable of promoting beneficial effects on wound healing in different models [13–15]. A group of secondary metabolites that have attracted much attention in recent years is triterpenes [16]. The pentacyclic triterpenoids belong to a class of C30 isoprenoids present in all parts of the plant, such as leaves, stems, pollen, seeds, and fruits [17]. They assist in wound healing mainly due to their inhibitory effects on the production and activity of inflammatory mediators and growth factors, resulting in the contraction of the wound and increased rate of epithelization [18].

Lupeol is a triterpene found in many medicinal plants, such as *Bowdichia virgilioides* (Fabaceae). Previous studies have reported that lupeol has several bioactivities, including antidiabetic, anti-inflammatory, and antioxidant effects [19, 20]. In particular, lupeol demonstrated an effect on cell proliferation in vitro through various mechanisms of action, including induction of differentiation [21] and activation of mitogen-activated protein kinase p38 (MAPK) [22] and phosphoinositide-3-kinase-protein kinase (Pi3k/Akt) [23]. These findings appear to be involved in the healing effects of lupeol. Harish et al. [24] showed that topical treatment with lupeol gel promoted cutaneous wound closure in normoglycemic rats by inducing the formation of granulation tissue, inhibiting the infiltration of macrophages, and increasing the reepithelialization. However, the efficacy of lupeol on hyperglycemia-induced impaired wound healing has not been investigated, and the mechanisms underlying these effects remain unknown. In the present study, we investigated whether lupeol enhances wound healing in streptozotocin-induced hyperglycemic rats via reduction of the inflammatory process and an increase in markers involved in oxidative stress, angiogenesis, formation of granulation tissue, and extracellular matrix remodelling.

## 2. Materials and Methods

### 2.1. Chemicals and Reagents

Streptozotocin (STZ) was obtained from Sigma-Aldrich Chemicals (St. Louis, MO, USA). Diagnostic kits for ELISA, such as TNF-*α*, IL-1*β*, IL-6, and IL-10, were bought from R&D Systems, Minneapolis, MN, USA. Primary antibodies specific to NF-*κ*B, TGF-*β*1, FGF-2, collagen III, and *β*-actin and anti-rabbit and/or anti-mouse secondary antibodies conjugated to horseradish peroxidase were attained from Santa Cruz Biotechnology (Santa Cruz, CA). All the chemicals used in the experiments were of analytical grade [3].

### 2.2. Plant Material, Extraction, and Isolation of Lupeol

*Bowdichia virgilioides* Kunth (stem bark) was collected in December 2014 in the surroundings of Santa Rita, State of Paraíba, Brazil, a coastal area around the Atlantic Forest. A voucher specimen (*Agra* et *Góis* 6243) was deposited at the Herbarium Prof. Lauro Pires Xavier (JPB) and in the reference collection of the Laboratory of Pharmaceutical Technology from Federal University of Paraíba, João Pessoa, Brazil. Three kilograms of air-dried ground stem bark of *Bowdichia virgilioides* was exhaustively extracted with 95% alcohol solution. The extracted solution was filtered, and the solvents were subjected to the evaporation method under reduced pressure with rotary evaporation at 40°C to obtain the final ethanolic extract (EtOHE, 250 g). The EtOHE was partitioned using solvents in increasing polarity (hexane, chloroform, and methanol). The hexane residue (49 g) was subjected to repeated washings with acetone under stirring followed by filtration. The solid obtained was recrystallized from chloroform and hexane, resulting in white crystals which were analyzed using ^1^H and ^13^C NMR spectral data and identified as lupeol substance (3 g) [23].

### 2.3. Animals

Healthy male *Wistar* rats (*Rattus norvegicus*) with body weight around 250 g ± 2 g were procured from Central Animal House, UNESP, Botucatu, and used for the study. The rats were housed individually in polyethylene cages in an experimental animal room with a 12 h light/dark cycle and maintained in standard conditions in the animal house at a room temperature of 23 ± 2°C and a humidity of 55%±15%. They were fed with a commercial standard rat diet and water *ad libitum*. 7 days prior to the start of the experiment, the rats were acclimatized to the animal house environment. All the experimental procedures were approved by the Ethics Committee on Animal Use (CEUA/IBB/UNESP), and the guidelines are followed strictly throughout the experimental period in accordance with the experimental protocols (Protocol 610/2014) previously approved.

### 2.4. Acute Dermal Irritation Test

Lupeol-based cream was used to evaluate skin irritation at concentration of 0.2% *w*/*w* lupeol as per OECD 402 directive. Healthy male *Wistar* rats weighing between 200 and 250 g were divided into two groups: 1 group treated with lupeol cream at 0.2% *w*/*w* and another group treated with Lanette cream (vehicle). After total removal of hairs in the dorsal region of the rats, treatments were performed over 14 days. The animals were observed twice daily for 14 days for any skin changes indicative of adverse reactions, such as irritation, edema, pruritus, and erythema, and some signs of toxicity, such as tremors, convulsions, salivation, diarrhea, sleep, and general pattern of behavior.

### 2.5. Induction of Experimental Hyperglycemia

Single intraperitoneal injection of streptozotocin (STZ) freshly prepared in citrate buffer (0.1 M, pH 4.5), at a concentration of 55 mg/kg body weight, was used to induce experimental hyperglycemia in overnight-fasted rats. After two days of induction, animals with blood glucose level ≥ 250 mg dL^−1^ were defined as hyperglycemic and kept under observation for another 7 days. Then, the hyperglycemic rats that maintained high glycemic levels were considered for the *in vivo* experiments. Furthermore, a weekly blood glucose test, body weight detection, and water and feed intake assessment were performed to ensure stable hyperglycemia status. This procedure followed the methodology described by Romana-Souza et al. [25].

### 2.6. Excision Wound Model and Experimental Design

The hyperglycemic rats that maintained high glycemic levels were considered for the wounding induction. All rats were anesthetized with ketamine hydrochloride (0.08 mg/100 g) and xylazine (0.04 mg/100 g) by intraperitoneal (IP) injection. Then, all the animals received a single dose of ketoprofen by subcutaneous (SC) injection (100 mg/kg) as an ethical conduct to minimize postoperative discomforts. Next, after shaving the hair on the back of each rat, the skin was sterilized with 70% alcohol to remove any type of contamination and a full-thickness excisional wound was created in the posterior dorsal region that extended through the *panniculus carnosus* of each animal using a 2 cm diameter punch. To assess the cutaneous wound healing of lupeol-based cream, the rats were randomly divided into four groups (*n* = 8), as shown below:

Group I Topically treated with Lanette cream (vehicle)
Group II Topically treated with insulin-based cream 0.5 U/g (positive control)
Group III Topically treated with 0.2% *w*/*w* lupeol cream (substance test)
Group IV Sham group (without diabetes, wounds, or treatment)

**Figure 1:**
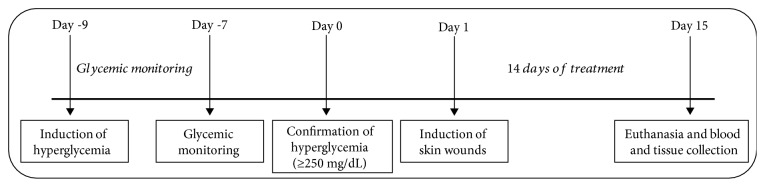
Overview of the experimental protocol for wound healing in streptozotocin-induced hyperglycemic rats.

Formulations were applied topically every day, once a day during 14 days of treatment. A schematic drawing of the experimental protocol is shown in Figure 1. The rats were placed in their respective cages with one rat per cage.

### 2.7. Collection of Blood Samples

After 14 days of experimentation, all the animals were anesthetized, and 5 mL of blood samples was collected into ethylenediaminetetraacetic acid- (EDTA-) embedded tubes and centrifuged and the serum was aspirated carefully for various biochemical assays, such as AST, ALT, *γ*-GT, alkaline phosphatase, creatinine, and urea.

### 2.8. Determination of Macroscopic Parameters

The wound area was measured daily through photographs with a professional camera and marked on a transparent sheet, and the surface area of the wound was measured. After scanning, the wound area was measured using specific software Adobe Photoshop C5 version 5 (Adobe Systems Inc., San Jose, California). The percentage of the wound area was calculated using the following formula: %retraction = {(initial lesion area − area of lesion on the day)/initial lesion area}∗100. The clinical signs of the lesions, such as exudation, presence of crust, and granulation tissue, were monitored by macroscopic examination and graded on a four-point scale: 0—absent (0%), 1—light (30%), 2—moderate (30-70%), and 3—intense (>70%) [26].

### 2.9. Histopathological Examination

The tissue samples from the skin were fixed with alcohol, formalin, and acetic acid and processed in paraffin. Skin tissues were sectioned (5 *μ*m). The inflammatory cell infiltration, proliferation of fibroblasts, and number of blood vessels were assessed by hematoxylin and eosin (HE) staining, and the deposition of collagen fibers was assessed by Masson's trichrome staining. For all analyses, two distinct regions were photographed: the border and the center of the wound. Ten photomicrographs of each sample were analyzed under a 40x magnification according to the method as previously described [27], being 5 for the border and 5 of the central lesion regions. All these parameters were quantified by the marked area count, totaling an area of 100.000 *μ*m^2^/slice. The photomicrographs were obtained with the software cellSens Standard (Olympus, USA), and the measurements were made using the AVSoft BioView software.

### 2.10. Immunohistochemistry Analysis

The immunohistochemical studies were performed with deparaffinized, rehydrated sections fixed on silanized slides and submitted to antigen recovery by pressure (20 psi/125°C). After washing, the sections were blocked with bovine serum albumin (BSA) and then incubated with primary antibodies against NF-*κ*B (1 : 100 *μ*L, Abcam, Cambridge, UK), FGF-2 (1 : 500 *μ*L, Abcam, Cambridge, UK), TGF-*β*1 (1 : 300 *μ*L, Abcam, Cambridge, UK), and collagen III (1 : 100 *μ*L, Abcam, Cambridge, UK) overnight at 4°C. The slices were incubated with appropriate biotinylated secondary antibodies. Then, the sections were stained with 3′-diaminobenzidine (DAB) and counterstained with hematoxylin. Finally, ten photomicrographs at 40x magnifications of each slice were analyzed with the software cellSens Standard (Olympus, USA), being 5 for the border and 5 for the central lesion regions [27]. The immunolabeled area was quantified totalizing 100.000 *μ*m^2^/slice. The quantification was made with the software AVSoft BioView.

### 2.11. Enzyme-Linked Immunosorbent Assay (ELISA)

The skin samples were homogenized in cold PBS supplemented with protease inhibitor cocktail (Sigma-Aldrich) and centrifuged at 10000 rpm for 20 min at 4°C, and then the supernatants were used to measure the cytokine levels of TNF-*α*, IL-1*β*, IL-6, and IL-10, as per the manufacturer's instructions from commercial enzyme-linked immunosorbent assay (ELISA) kits (R&D Systems, Minneapolis, MN, USA) [3]. Total proteins were assessed by the biuret assay so that the data of the parameters analyzed are expressed relative to the amount of protein in the sample in milligrams [28].

### 2.12. RNA Extraction and RT-qPCR

The total RNA of the skin samples (*R. norvegicus*) was extracted using the TRIzol method (Invitrogen, Carlsbad, CA, USA), following the manufacturer's recommendations and with adaptations, according to Nóbrega et al. [29]. To avoid genomic DNA contamination, the RNA samples were treated with a DNase I- and RNase-free kit (Invitrogen, Carlsbad, CA, USA) prior to cDNA synthesis. The cDNA synthesis was performed with random hexamers using SuperScript® II (Invitrogen, Carlsbad, CA, USA) with random hexamers according to standard protocols. The quantitative PCR reaction was carried out using designed and specific forward and reverse primers for *R. norvegicus* (Table 1) to evaluate the expression of the genes for *Nf-κb*, *Ki-67*, *Egf*, *Vegf-A*, *Ho-1*, *Ho-2*, *Gpx-1*, *Sod-2*, *Hif-1α*, *Nos-2*, *Angpt-4*, and *Col3α1* among the different treatments used. For qPCR, Ct values were determined using the SYBR Green kit (Invitrogen, Carlsbad, CA, USA). All qPCR reactions (10 *μ*L) used 900 nM for each primer and 700 ng of total RNA. Each reaction was performed in duplicate in the StepOne System (Life Technologies, Carlsbad, CA, USA) following the manufacturer's instructions, and relative gene expression profiles were calculated, according to the ΔΔCt method as previously described [30]. And for each gene of interest, the mRNA levels (Cts) were normalized by the reference gene, *β-actin* (Table 1), and expressed with values relative to the Ct mean (a point from which the system starts the quantification of the genetic material from the exponential phase threshold) of each group (ddCt—Ct normalized by means of the respective *β-actin* groups).

### 2.13. Statistical Analysis

Statistical analysis was performed using GraphPad Prism (version 5.1, GraphPad Software Inc., San Diego, CA, USA). All the parametric data were expressed as mean ± standard error of the mean, and the comparison between groups was performed by ANOVA followed by the Newman-Keuls test. The nonparametric data were expressed as median (maximum and minimum), and the comparison was performed by the Kruskal-Wallis test, followed by the Dunn test. The values of *p* < 0.05 were considered statistically significant.

## 3. Results

### 3.1. Acute Dermal Irritation

Topical application of lupeol treatment at 0.2% *w*/*w* did not show any adverse reactions on rat skin, i.e., irritation, edema, pruritus, and erythema, as compared to the control group. There were also no observed signs of toxicity or behavioral changes.

### 3.2. Hyperglycemia Status

All rats used in the present study showed the characteristic signs of hyperglycemia from the second day after the administration of STZ. Blood glucose levels increased significantly in all groups after 48 hours of streptozotocin administration and remained elevated throughout the experiment (Table 2). All other signs are described in supplementary materials. These include weight loss (Table S1), polydipsia (Table S3), and polyphagia (Table S4). Serum levels of aspartate aminotransferase (AST), alanine aminotransferase (ALT), *γ*-glutamyl transferase (GGT), alkaline phosphatase, and urea were measured after 14 days of experimentation and showed significant changes in relation to the sham group (Table S2).

### 3.3. Wound Closure

**Figure 2:**
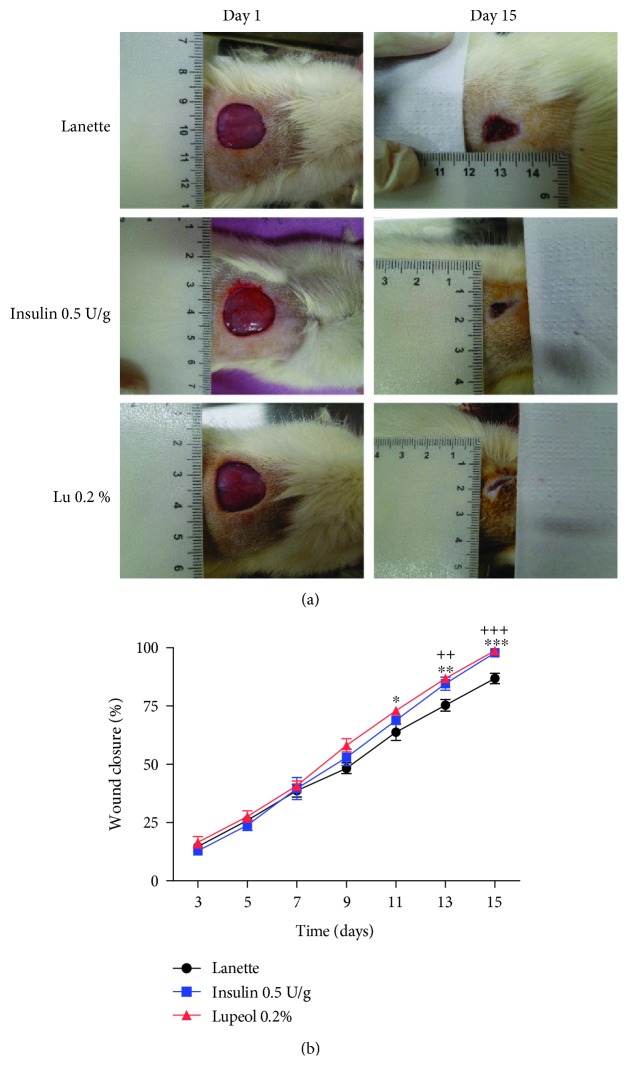
Effect of lupeol on skin wound healing in streptozotocin-induced hyperglycemic rats. Representative images of the lesion area (initial and from the last day of experimentation) of animals treated with Lanette, insulin 0.5 U/g, and lupeol 0.2% (a) and wound closure (%) on days 3, 5, 7, 9, 11, 13, and 15 postwounding (b). ^∗^ *p* < 0.05, ^∗∗^ *p* < 0.01, and ^∗∗∗^ *p* < 0.001 represent lupeol vs. Lanette group. ^++^ *p* < 0.01 and ^+++^ *p* < 0.001 represent insulin vs. Lanette group, using ANOVA followed by the Newman-Keuls test. Lu 0.2% = lupeol 0.2%.

The evaluation of the lesion area revealed that the topical application of lupeol-based cream notably decreased wound size (Figure 2(a)), with a significant increase in the percentage of lesion retraction in relation to Lanette (Figure 2(b)). The mean percentage of lesion retraction was markedly higher in the lupeol-treated group from day 11, and this difference remained until day 15 at termination. The insulin-treated group showed a significant increase in the percentage of wound contraction on days 13 and 15 compared to the control group. Clinical parameters, such as coagulation, presence of crust, and granulation tissue, were analyzed in all rats. The macroscopic observations after 14 days of treatment showed that some lesions still presented little clot and granulation tissue. Lanette treatment still had a significant amount of crust adhered to the lesion, compared to the lupeol-treated group, which did not present this crust, showing only the scar of the injured region (Table 3).

### 3.4. HE Staining

**Figure 3:**
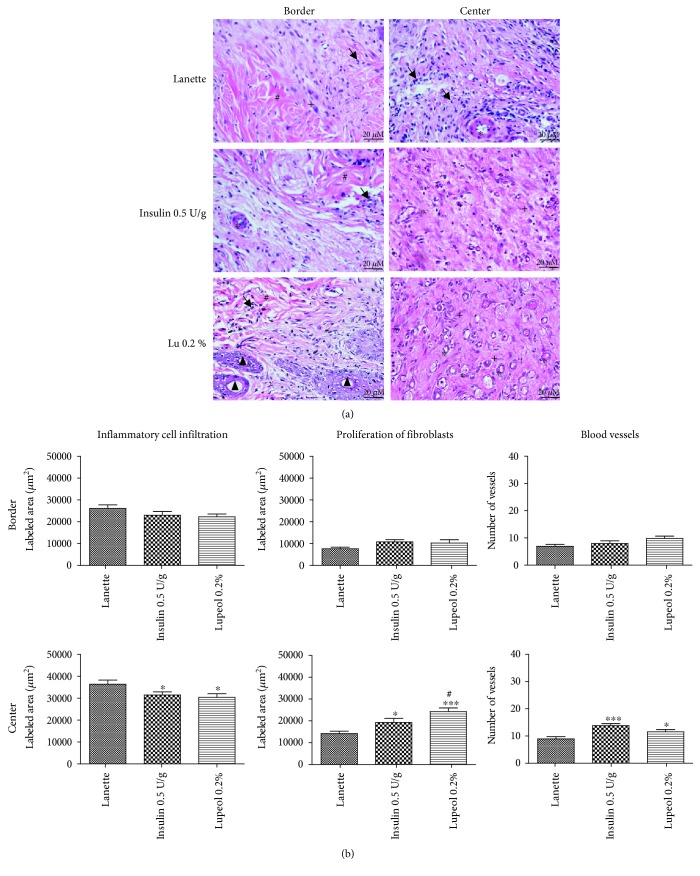
HE-stained skin tissue sections on day 14 postwound induction in streptozotocin-induced hyperglycemic rats (a). Inflammatory cell infiltration, proliferation of fibroblasts, and number of blood vessels (b) in HE staining of the border and central region of rats' hyperglycemic wounds treated with Lanette, insulin 0.5 U/g, or lupeol 0.2% for 14 days. ^∗^ *p* < 0.05 and ^∗∗∗^ *p* < 0.001 vs. Lanette group. ^#^ *p* < 0.05 vs. insulin group, using ANOVA followed by the Newman-Keuls test. Bar represents 20 *μ*m. Black arrows indicate the presence of inflammatory cells, # indicates collagen fibers, ^∗^ indicates blood vessels, + indicates fibroblasts, and ▲ indicates sebaceous glands. Lu 0.2% = lupeol 0.2%.

Representative images of HE-stained wound sections are presented in Figure 3(a), and the number of blood vessels, inflammatory cell infiltration, and proliferation of fibroblasts in the border and the lesion center are presented in Figure 3(b). We observed a significant reduction in inflammatory cell infiltration in the lupeol- and insulin-treated groups but not in the Lanette group (Figure 3(b)). Lupeol treatment also significantly increased fibroblast proliferation compared to Lanette-and insulin-treated groups (Figure 3(b)). Both lupeol and insulin treatments significantly increased the number of blood vessels in the center of the lesions in relation to Lanette (Figure 3(b)).

### 3.5. Masson's Trichrome Staining

**Figure 4:**
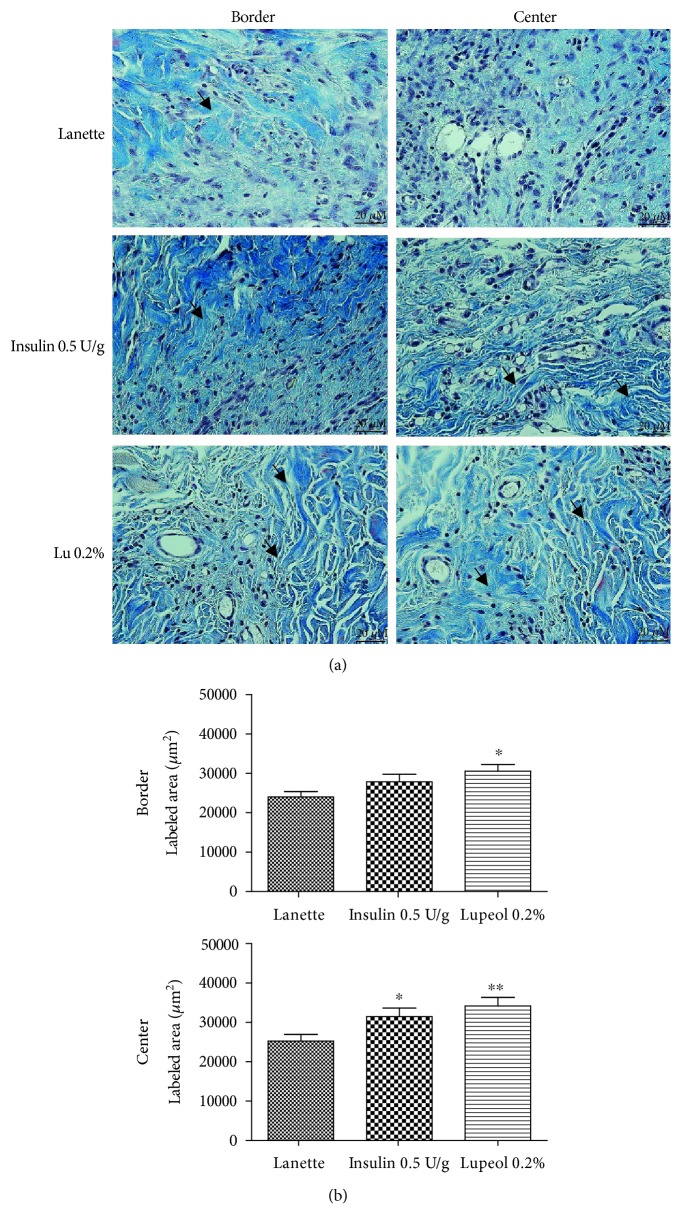
Masson's trichrome-stained skin tissue sections on day 14 postwound induction in streptozotocin-induced hyperglycemic rats (a). Labeled area of total collagen fibers (b) (*μ*m^2^) in the border and central region of rats' hyperglycemic wounds treated with Lanette, insulin 0.5 U/g, or lupeol 0.2% for 14 days. ^∗^ *p* < 0.05 and ^∗∗^ *p* < 0.01 vs. Lanette group, using ANOVA followed by the Newman-Keuls test. Bar represents 20 *μ*m. Black arrows indicate the presence of total collagen fibers. Lu 0.2% = lupeol 0.2%.

Analysis of total collagen fibers using Masson's trichrome method showed the labeled area (*μ*m^2^) in the border and center regions of the lesion as shown in Figure 4(a). The deposition, orientation, and organization of collagen fibers were considerably clearer in lupeol-treated wounds than in the other groups (Figure 4(b)). Lupeol treatment caused a significant increase in the area labeled by collagen fibers in the border and the center of the lesion compared to Lanette (Figure 4(b)).

### 3.6. Effect of Lupeol on Immunolabeling of NF-*κ*B, TGF-*β*1, FGF-2, and Collagen III

**Figure 5:**
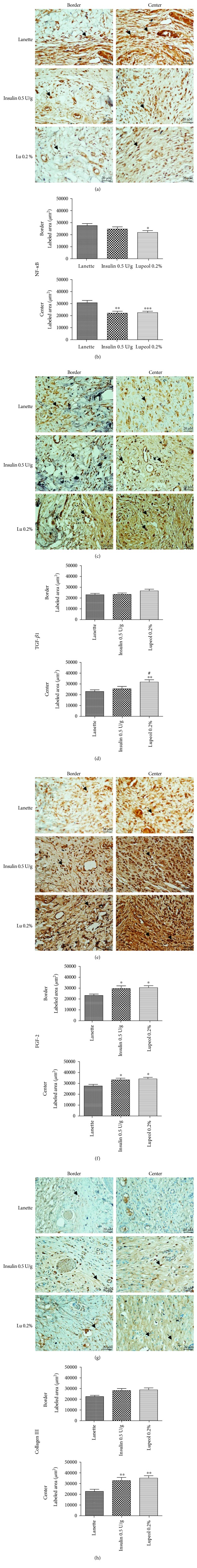
Photomicrography of the immunostaining and immunolabeled area (*μ*m^2^) for NF-*κ*B (a, b), TGF-*β*1 (c, d), FGF-2 (e, f), and collagen III (g, h) in the border and central region of rats' hyperglycemic wounds treated with Lanette, insulin 0.5 U/g, or lupeol 0.2% for 14 days. ^∗^ *p* < 0.05, ^∗∗^ *p* < 0.01, and ^∗∗∗^ *p* < 0.001 vs. Lanette group. ^#^ *p* < 0.05 vs. insulin group, using ANOVA followed by the Newman-Keuls test. Bar represents 20 *μ*m. Black arrows indicate antibody staining against NF-*κ*B, TGF-*β*1, FGF-2, and collagen III. Lu 0.2% = lupeol 0.2%.

Representative images of the immunohistochemistry of NF-*κ*B, TGF-*β*1, FGF-2, and collagen III in experimental wounds are shown in Figures 5(a), 5(c), 5(e) and 5(g). Lupeol treatment significantly reduced the NF-*κ*B immunolabeling in the lesion border and center in relation to Lanette treatment (Figure 5(b)). With immunolabeling to localize TGF-*β*1, lupeol treatment showed a significant increase in the antibody in the wound center in relation to Lanette- and insulin-treated groups, as shown in Figure 5(d). Regarding FGF-2, there was a significant increase in immunolabeling of both the border and the lesion center treated with lupeol and insulin cream compared to Lanette treatment (Figure 5(f)). Lupeol also significantly increased the collagen III-immunolabeled area in the central region of the lesion as compared to the Lanette group (Figure 5(h)).

### 3.7. Effect of Lupeol on Pro- and Anti-Inflammatory Markers

**Figure 6:**
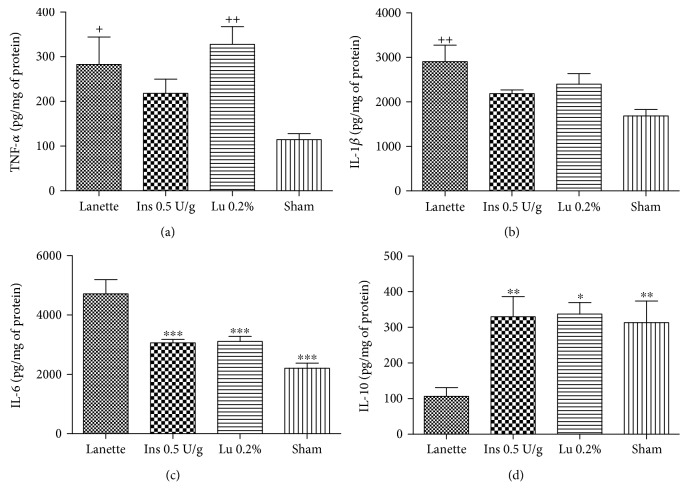
Quantification of TNF-*α* (a), IL-1*β* (b), IL-6 (c), and IL-10 (d) levels (pg/mg protein) in rats' hyperglycemic wounds treated with Lanette, insulin 0.5 U/g, or lupeol 0.2% for 14 days. ^∗^ *p* < 0.05, ^∗∗^ *p* < 0.01, and ^∗∗∗^ *p* < 0.001 vs. Lanette group. ^+^ *p* < 0.05 and ^++^ *p* < 0.01 vs. sham group, using ANOVA followed by the Newman-Keuls test. Lu 0.2% = lupeol 0.2%.

**Figure 7:**
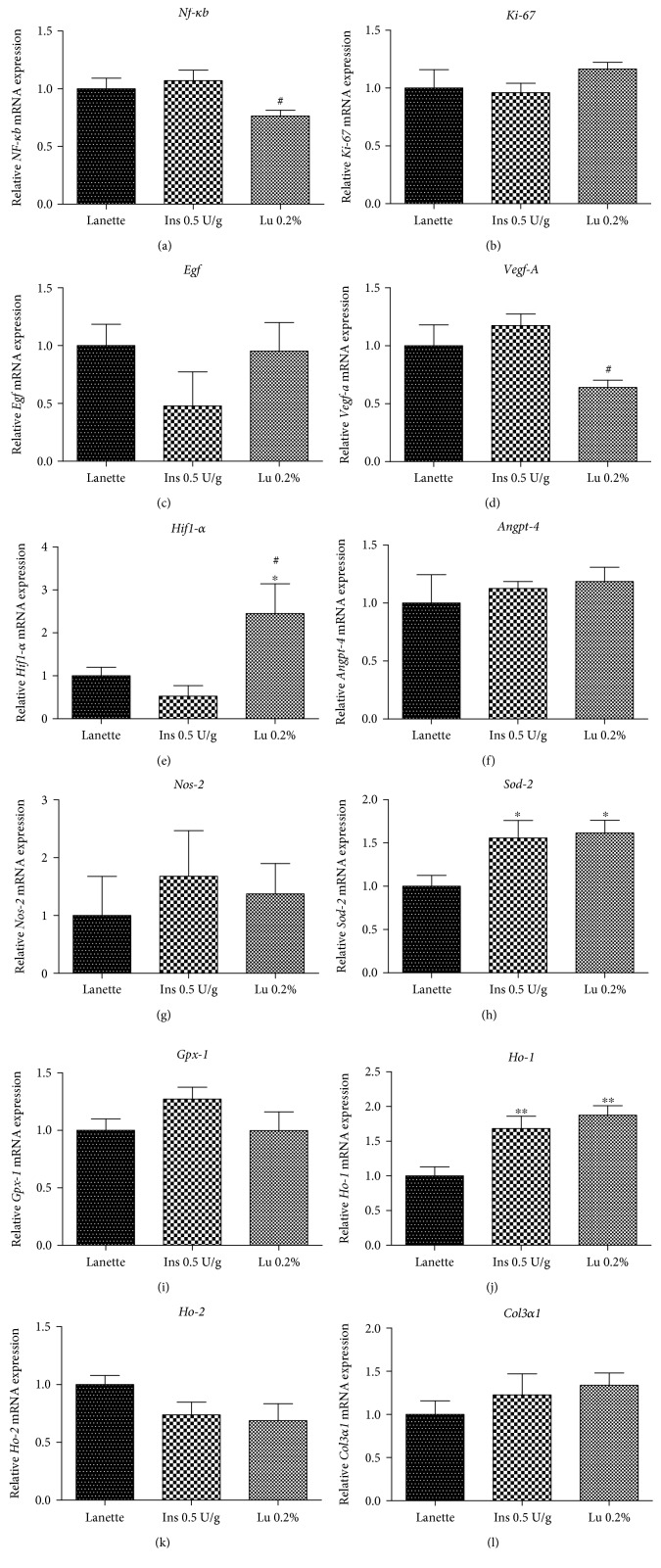
Gene expression (RT-qPCR) of *Nf-κb* (a), *Ki-67* (b), *Egf* (c), *Vegf-A* (d), *Hif-1α* (e), *Angiopoietin-4* (f), *Nos-2* (g), *Sod-2* (h), *Gpx-1* (i), *Ho-1* (j), *Ho-2* (k), and *Col3α1* (l) in rats' hyperglycemic wounds treated with Lanette, insulin 0.5 U/g, or lupeol 0.2% for 14 days. ^∗^ *p* < 0.05 and ^∗∗^ *p* < 0.01 vs. Lanette group. ^#^ *p* < 0.05 vs. insulin group, using ANOVA followed by the Newman-Keuls test. Lu 0.2% = lupeol 0.2%.

The results obtained through ELISA showed that lupeol treatment caused a significant increase in TNF-*α* levels when compared to the sham group (Figure 6(a)). In contrast, there was a significant reduction of IL-6 levels in the insulin- and lupeol-treated groups in relation to the Lanette-treated group (Figure 6(c)). IL-1*β* levels were significantly increased only in the Lanette-treated group as compared to the sham group (Figure 6(b)). However, IL-10 levels remained significantly elevated in the insulin-treated and lupeol groups relative to the Lanette group (Figure 6(d)). mRNA expression of proinflammatory mediators demonstrated a significant increase in the *Nf-κb* expression in the insulin-treated group as compared to lupeol treatment and no significant change in the mRNA expression of *Nos-2*, as shown in Figures 7(a) and 7(g), respectively. These results provide evidence for lupeol having anti-inflammatory potential by reducing *Nf-κb* and IL-6 and increasing IL-10 levels.

### 3.8. Effect of Lupeol on Molecular Markers of Angiogenesis and Cell Proliferation

The mRNA expression of *Vegf-A*, *Hif-1α*, and *Angiopoietin-4* as markers of the angiogenic process in wound healing was evaluated. Lupeol treatment caused a reduction in *Vegf-A* expression compared to insulin treatment (Figure 7(d)). On the other hand, lupeol effectively stimulated a significant increase in *Hif-1α* expression compared to the Lanette- and insulin-treated groups, as shown in Figure 7(e). No significant change in *Angiopoietin-4* expression could be observed (Figure 7(f)). There were also no significant changes in the expression of *Ki-67*, *Egf*, or *Col3α1*, markers involved in the proliferative wound healing phase (Figures 7(b), 7(c), and 7(l)).

### 3.9. Effect of Lupeol on Molecular Markers of Oxidative Stress

Analyses of markers such as *Ho-1*, *Ho-2*, *Gpx-1*, and *Sod-2* related to oxidative stress through real-time gene expression were performed. The results showed that the lupeol and insulin treatment significantly increased the expression of *Sod-2* and *Ho-1* compared to vehicle (Figures 7(h) and 7(j)). There was no significant change in the expression of *Gpx-1* and *Ho-2* among the treated groups, as shown in Figures 7(i) and 7(k).

## 4. Discussion

We have demonstrated a role for lupeol, a pentacyclic triterpene, in wound healing in streptozotocin-induced hyperglycemic rats. The results showed markedly decreased expression of *NF-κB* and IL-6 and increased IL-10 levels in the lupeol-treated group compared to the control group. Lupeol also increased the expression of FGF-2, TGF-*β*1, *Hif-1α*, *Ho-1*, and *Sod-2*. In addition, we have histologically observed a better formation of granulation tissue with marked proliferation of fibroblasts, increased vascularization, and deposition of collagen fibers after lupeol treatment. The results suggest a better and accelerated wound healing in hyperglycemic rats treated with lupeol.

Impaired wound healing represents one of the major diabetic complications in clinical practice. In general, hyperglycemia causes changes in the functioning of endothelial cells and, consequently, vascular dysfunction in the wounds, which become infected and, in many cases, lead to amputations [31]. It is well known that changes in several endogenous factors contribute to the delayed wound healing, including low growth factor production [32], mediators involved in the angiogenic response [33, 34], macrophage function [33], collagen synthesis, epidermal barrier function, keratinocyte migration, and fibroblast proliferation [32]. The healing process has been documented animal models, and excisional wound models have become effective methods of preclinical testing in diabetic rat studies [35]. Rodents are commonly used as a wound healing model. However, the mechanics of wound healing differ between rodents and humans. In humans, wound healing primarily depends on keratinocyte growth and migration in the epidermis and granulation tissue formation in the dermis, whereas wound contraction is more important during rodent wound closure. This is because rodents possess a subcutaneous panniculus carnosus muscle that facilitates skin healing by both wound contraction and collagen formation [36]. Nonetheless, human wounds depend on the contractile properties of dermal (myo)fibroblasts, especially during the remodelling phase of healing [37].

Measurement of blood glucose levels is one of the most effective diagnostic methods in monitoring diabetes. Thus, glycemic evaluation was chosen as the main parameter for disease progression in this study. A single injection of STZ was able to significantly increase the glycemia of fasted rats. This is because STZ is a diabetogenic agent, inducing necrosis by damaging pancreatic *β*-cell DNA [38] with an effective decrease in insulin production, which eventually generates a diabetic phenotype. In the present study, all animals after STZ injection maintained blood glucose levels above 250 mg/dL, even after any of the topical treatments, suggesting that they did not interfere with the blood glucose levels of the animals throughout the experimental period. Previous studies have also reported similar observations, with effective wound healing activity in rats without decreasing glycemia of these animals [39, 40]. In our study, characteristic signs were noted from the third day of STZ administration. We observed a decrease in body weight gain, polyphagia, polydipsia, and polyuria in all animals, which according to the literature are the main clinical manifestations of diabetes [41, 42].

Studies conducted through clinical trials indicated that the cutaneous wound healing process occurs in three phases: the inflammatory phase due to high release of proinflammatory mediators and impairment of the immune system, the proliferative phrase through the proliferation of fibroblasts, collagen fiber deposition, and formation of new blood vessels, and the remodelling phase involving restoration and repair of injured tissue [43, 44]. For a successful therapy, it would require a drug that accelerates the wound healing with potential involvement in all phases of the process, with low cost and less side effects. The findings of the present study showed that lupeol was effective in promoting skin wound healing in hyperglycemic rats, by reducing the lesion size from day 11, accompanied by wound contraction. Our results are in accordance with Harish et al. [24] who showed that lupeol-based gel enhanced skin wound healing in normoglycemic rats through excision, incision, and dead space wound models.

It is known that diabetic wounds show exacerbated production of inflammatory cells at wound sites, which may limit wound closure efficacy [45]. Our data showed a reduction of inflammatory cells in the wound site of diabetic rats treated with lupeol-based cream, specifically in the central region of the lesion. Chronic inflammation is also one of the main factors contributing to the delayed wound healing. Previous studies have shown that hyperglycemic wounds are capable of causing a decrease in the expression of anti-inflammatory cytokines, such as IL-10 and TGF-*β*, and an increase in the expression of proinflammatory cytokines, such as TNF-*α*, IL-1*β*, and IL-6, as well as inflammatory cells [46, 47]. However, despite the fact that they are involved in the inflammatory process, proinflammatory cytokines are also considered mediators of cell proliferation and differentiation during the wound healing process [48]. For instance, IL-6 stimulates the formation of granulation tissue, reepithelialization mechanisms, and angiogenesis when activated by keratinocytes [49]. TNF-*α* shows a dual role in decreasing granulation tissue formation and collagen fiber arrangement [50, 51]. In the present study, lupeol treatment favorably regulated these mediators by reducing proinflammatory cytokines such as IL-6 and by upregulating the levels of IL-10, an anti-inflammatory cytokine.

In addition to cytokines, studies report the involvement of NF-*κ*B with chronic inflammation in diabetic wounds. Once activated, NF-*κ*B translocates from the cytoplasm to the nucleus of the cell, where it binds to DNA and promotes a transcription of several proinflammatory mediators, including NOS-2 or iNOS gene expression [52]. It is known that overproduction of iNOS during hyperglycemia is correlated with prolonged inflammation within the dermis and delays wound healing [53]. The results of the present study showed that topical application of lupeol reduced the expression of NF-*κ*B, but not iNOS expression, and enhanced wound healing by preventing prolonged inflammation. Our data are similar to the data of a recent study [23], which showed that lupeol promoted wound healing in human epidermal keratinocytes via anti-inflammatory mechanisms through inhibition of proinflammatory mediators such as NF-*κ*B. Taken together, these results indicate an anti-inflammatory effect of lupeol in hyperglycemic wounds.

A persistent inflammatory state through high infiltration of inflammatory cells into the wound site also produces oxidative stress by generating several reactive oxygen species (ROS) and reactive nitrogen species, capable of producing various cytotoxic effects on cells [54, 55]. SOD and CAT are the natural cellular antioxidants that can play a crucial role in oxygen defense metabolism by converting superoxide ions to water and molecular oxygen [56]. Another essential enzyme expressed under conditions of elevated oxidative stress is HO-1 [57]. Studies report that, in addition to its potent antioxidant effect, HO-1 also presents proangiogenic and anti-inflammatory properties [58, 59]. Our results obtained evidenced a significant increase in the gene expression of both enzymes, *Sod-2* and *Ho-1*, in lupeol- and insulin-treated groups, suggesting an antioxidant effect by neutralizing the action of the free radicals generated in the wound.

Wound healing quality depends on the granulation tissue formation that is directly affected in diabetics. An increase in granulation tissue formation requires an increase in cell proliferation of fibroblasts and endothelial cells and increased collagen synthesis [60, 61]. It is well documented that collagen is one of the major and most important components of granulation tissue, and its synthesis occurs in fibroblasts, which is dependent on TGF-*β*1, a crucial factor for proper wound healing [62]. The role of lupeol in the proliferative phase was validated through histopathological studies, where it was possible to observe an increase in the proliferation of fibroblasts in the central region of the wounds, an increase in the formation of collagen fibers in both the borders and the center of the lesion, and elevated immunolabeling of collagen type III in the wound center.

Fibroblasts play a predominant role during the wound contraction process [63]. Wound contraction is a fundamental phenomenon in the complete wound closure and is associated with differentiated myofibroblasts, specialized cells present in granulation tissue [64]. FGFs (fibroblast growth factors) stimulate the proliferation and migration of various cells involved in wound healing. They also stimulate collagen synthesis, epithelialization, and neovascularization [65]. FGF-2 is one of the most important growth factors of the proliferative phase, produced mainly by keratinocytes, and stimulates proliferation and migration of fibroblasts, new blood vessel formation, and ECM production [66]. Our results showed a strong expression of FGF-2 in the border and center of hyperglycemic lesions treated with lupeol.

Angiogenesis is important for the maintenance of tissue health and quicker wound healing. Impairment in new blood vessel formation delays the healing process and induces severe ulcerations. Moreover, cutaneous blood flow and neovasculogenesis are impaired in diabetes, which in turn affects the healing process of wounds. Improved angiogenesis was demonstrated in our findings through histopathological analyses in the central region of the lesions after treatment with lupeol cream. Numerous molecules, growth factors, and cytokines are involved in regulating the angiogenesis, such as VEGF, FGF-2, and HIF-1*α* [67]. HIF-1*α* has a crucial role in, since it is necessary for the expression of multiple angiogenic growth factors, cell motility and recruitment of endothelial progenitor cells [68]. In our study, increased expression of TGF-*β*1and *Hif-1α* in the lupeol-treated group possibly explains the formation of new vessels observed in histopathological analyses.

In the present study, as expected, the application of the insulin-based cream produced some beneficial effects compared to the control. Our data are consistent with the literature [69] that showed an improvement in diabetic wound healing by insulin-based cream. Insulin treatment caused an increase in the percentage of wound contraction from the 13th day, while the lupeol had an effect from the 11th day. Although most effects on molecule expression showed similarities to the lupeol-treated group, insulin treatment differed by not increasing the levels of TGF-*β*1 and *Hif-1α*, crucial mediators in the angiogenic process.

**Figure 8:**
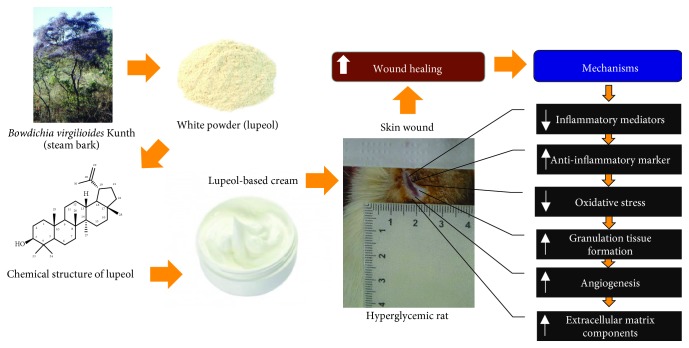
Schematic representation of the regulatory mechanisms of lupeol-based cream in wound healing of hyperglycemic rats.

Overall, the study demonstrated through an excisional wound model in streptozotocin-induced hyperglycemic rats that topical application of lupeol accelerates cutaneous wound healing by promoting anti-inflammatory and antioxidant effects, neovascularization, and granulation tissue formation (Figure 8). Thus, lupeol, a pentacyclic triterpene, isolated from the bark of stem of *B. virgilioides* might be considered a therapeutic agent for wounds in patients with diabetes.

## 5. Conclusion

Wound treatment using lupeol-based cream effectively enhanced the healing process in hyperglycemic rats through the anti-inflammatory effect of NF-*κ*B signaling pathways, as suggested by a reduction of inflammation-associated mediators, such as IL-6 (proinflammatory cytokine), and elevated IL-10 levels (anti-inflammatory cytokine). Histopathological findings also revealed a decrease in the inflammatory process and faster neovasculogenesis and proliferation of fibroblasts in the lupeol-treated group. The involvement of *Hif-1α*, FGF-2, and TGF-*β*1, crucial to the angiogenesis process as well as fibroblast infiltration, proliferation, and migration in wound sites, efficiently mediated the injury and returned the wound to its original state. In addition to this, lupeol also minimized the oxidative stress and improved the antioxidant status through increased mRNA expression of *Ho-1* and *Sod-2*. This study provided us with good scientific evidence that the application of lupeol cream is promising for treating wounds in hyperglycemic rats.

## Figures and Tables

**Table 1:** Sequence of primers used in RT-qPCR.

Gene	Primer sequence 5′-3′	Product size	Melting temperature	Access number^∗^
*β-actin*	FW: CCCTGGCTCCTAGCACCAT	80 bp	60°C	NM_031144.3
	RV: GATAGAGCCACCAATCCACACA			
*Nf-κb*	FW: CCTCATCTTTCCCTCAGAGCC	98 bp	60°C	NM_199267.2
	RV: CGCACTTGTAACGGAAACGC			
*Ki-67*	FW: GGGTTTCCAGACACCAGACC	100 bp	60°C	NM_001271366.1
	RV: CCAGGAAGACCAGTTAGAACC			
*Egf*	FW: CTCAGGCCTCTGACTCCGAA	93 bp	60°C	NM_012842.1
	RV: ATGCCGACGAGTCTGAGTTG			
*Vegf-A*	FW: TGCGGATCAAACCTCACCAA	115 bp	60°C	NM_001110333.2
	RV: GGCTCACAGTGATTTTCTGGC			
*Ho-1*	FW: GTCCCAGGATTTGTCCGAGG	133 bp	60°C	NM_012580.2
	RV: GGAGGCCATCACCAGCTTAAA			
*Ho-2*	FW: CCGGGCAGAAAATACCCAGT	192 bp	60°C	NM_J05405.1
	RV: ATCAGTGCTTCCTTCCGGTG			
*Gpx-1*	FW: CATTGAGAATGTCGCGTCCC	141 bp	60°C	NM_030826.4
	RV: TTGCCATTCTCCTGATGTCCG			
*Sod-2*	FW: GTGGAGAACCCAAAGGAGAGT	177 bp	60°C	NM_017051.2
	RV: GGTCCTGATTAGAGCAGGCG			
*Hif1-α*	FW: ATCCATTTTCAGCTCAGGACAC	182 bp	60°C	NM_AF057308.1
	RV: GGTAGGTTTCTGTAACTGGGTCTG			
*Nos-2*	FW: GCCTAGTCAACTACAAGCCCC	87 bp	60°C	NM_012611.3
	RV: AGAAACTTCCAGGGGCAAGC			
*Angpt-4*	FW: AACTGTTCCAGAAGGTAGCCC	90 bp	60°C	NM_199115.2
	RV: TCAAGAGGTCAATCTGGCTCTG			
*Col3α1*	FW: GGGATCCAATGAGGGAGAAT	128 bp	60°C	NM_032085.1
	RV: CCTTGCGTGTTTGATATT			

FW: forward; RV: reverse; bp: base pairs. ^∗^National Center for Biotechnology Information (NCBI) (Nucleotide, https://www.ncbi.nlm.nih.gov).

**Table 2:** Blood glucose levels in different groups before and after administration (i.p.) of streptozotocin (STZ). ^∗∗∗^ *p* < 0.001 vs. sham group, using ANOVA followed by the Newman-Keuls test.

Groups	Day -9 (STZ injection)	Day -7 (post-STZ)	Day 0 (before wounding)	Day 8 (postwounding)	Day 15 (euthanasia)
Lanette	104.04 ± 3.93	498.12±14.02^∗∗∗^	482.53±11.37^∗∗∗^	543.09±8.85^∗∗∗^	530.61±21.37^∗∗∗^
Insulin 0.5 U/g	108.12 ± 4.21	501.09±23.87^∗∗∗^	524.84±19.99^∗∗∗^	501.67±15.4^∗∗∗^	511.36±11.87^∗∗∗^
Lupeol 0.2%	101.43 ± 5.80	476.89±16.29^∗∗∗^	498.89±14.82^∗∗∗^	554.03±25.8^∗∗∗^	543.08±27.32^∗∗∗^
Sham	104.45 ± 4.73	112.04 ± 6.91	109.26 ± 7.02	99.85 ± 8.853	113.93 ± 9.24

**Table 3:** Clinical parameters of hyperglycemic cutaneous wound after topical treatment with Lanette, insulin 0.5 U/g, and lupeol 0.2 % (*n* = 8) in rats. Data are expressed as median (minimum, maximum) and analyzed by the Kruskal-Wallis test, followed by the Dunn test. ^∗^ *p* < 0.05.

Parameters	Groups	Days after wound induction (14 days)
Coagulation	Lanette	0 (0, 2)
	Insulin 0.5 U/g	0 (0, 1)
	Lupeol 0.2%	0 (0, 1)

Presence of crust	Lanette	1 (0, 2)
	Insulin 0.5 U/g	0 (0, 1)
	Lupeol 0.2%	0 (0, 1)^∗^

Granulation tissue	Lanette	1 (0, 2)
	Insulin 0.5 U/g	0 (0, 1)
	Lupeol 0.2%	0 (0, 2)

## Data Availability

The data used to support the findings of this study are available from the corresponding author upon request.

## Supplementary Materials

Supplementary Materials Supplementary data are all other signs, such as weight loss, polydipsia, and polyphagia and biochemical parameters. Table S1: body weight evaluation of rats treated with Lanette, insulin 0.5 U/g, or 0.2% lupeol creams for 14 days of experimentation. Table S2: biochemical parameters of rats treated with Lanette, insulin 0.5 U/g, or lupeol 0.2% for 14 days of experimentation. Table S3: water intake (mL) in hyperglycemic rats treated with Lanette, insulin 0.5 U/g, or lupeol 0.2% during 14 days of experimentation. Table S4: food intake (g) in hyperglycemic rats treated with Lanette, insulin 0.5 U/g, or lupeol 0.2% during 14 days of experimentation.
