# Corrigendum to “Lupeol, a Dietary Triterpene, Enhances Wound Healing in Streptozotocin-Induced Hyperglycemic Rats with Modulatory Effects on Inflammation, Oxidative Stress, and Angiogenesis”

**DOI:** 10.1155/2020/3252696

**Published:** 2020-05-27

**Authors:** Fernando Pereira Beserra, Ana Júlia Vieira, Lucas Fernando Sérgio Gushiken, Eduardo Oliveira de Souza, Maria Fernanda Hussni, Carlos Alberto Hussni, Regina Kiomi Takahira, Rafael Henrique Nóbrega, Emanuel Ricardo Monteiro Martinez, Christopher John Jackson, Gabriela Lemos de Azevedo Maia, Ariane Leite Rozza, Cláudia Helena Pellizzon

**Affiliations:** ^1^Department of Morphology, Institute of Biosciences, São Paulo State University (UNESP), Botucatu, São Paulo, Brazil; ^2^Department of Surgery and Veterinary Anesthesiology, School of Veterinary Medicine and Animal Science, São Paulo State University (UNESP), Botucatu, São Paulo, Brazil; ^3^Kolling Institute of Medical Research, The University of Sydney at Royal North Shore Hospital, Sydney, Australia; ^4^Department of Pharmacy, Federal University of São Francisco Valley (UNIVASF), Petrolina, Pernambuco, Brazil

In the article titled “Lupeol, a Dietary Triterpene, Enhances Wound Healing in Streptozotocin-Induced Hyperglycemic Rats with Modulatory Effects on Inflammation, Oxidative Stress, and Angiogenesis” [1], Dr. Regina Kiomi Takahira was missing from the authors' list. Dr. Takahira conducted biochemical assays such as AST, ALT, *γ*-GT, alkaline phosphatase, creatinine, and urea, as well as the interpretation and visualization of all these data. The corrected authors' list is shown above.
