# Supplementary material for: Lupeol, a Dietary Triterpene, Enhances Wound Healing in Streptozotocin-Induced Hyperglycemic Rats with Modulatory Effects on Inflammation, Oxidative Stress, and Angiogenesis
Source: Oxid Med Cell Longev. 2019 May 9;2019:3182627. doi: 10.1155/2019/3182627 (PMC6532325; doi:10.1155/2019/3182627)
Supplement: Supplementary Materials — Supplementary data are all other signs, such as weight loss, polydipsia, and polyphagia and biochemical parameters. Table S1: body weight evaluation of rats treated with Lanette, insulin 0.5 U/g, or 0.2% lupeol creams for 14 days of experimentation. Table S2: biochemical parameters of rats treated with Lanette, insulin 0.5 U/g, or lupeol 0.2% for 14 days of experimentation. Table S3: water intake (mL) in hyperglycemic rats treated with Lanette, insulin 0.5 U/g, or lupeol 0.2% during 14 days of experimentation. Table S4: food intake (g) in hyperglycemic rats treated with Lanette, insulin 0.5 U/g, or lupeol 0.2% during 14 days of experimentation. [file 3182627.f1.docx]

**SUPPLEMENTARY MATERIALS**

| **Body weight (g)** | **Lanette** | **Insulin 0,5 U/g** | **Lupeol 0,2%** | **Sham** |
| --- | --- | --- | --- | --- |
| Initial weight | 276,6 ± 10,56 | 258,9 ± 6,479 | 285,6 ± 13,27 | 279 ± 7,125 |
| Final weight | 242,7 ± 10,60*** | 244,6 ± 5,961*** | 253,7 ± 12,91*** | 294,9 ± 44,27 |

**Table S1:** Body weight evaluation of rats treated with lanette, insulin 0.5 U/g or 0.2% lupeol creams for 14 days of experimentation (n=8). ****p* <0.001 vs. sham group, using ANOVA followed by the Newman-Keuls test.

|  |  |  |  |  |
| --- | --- | --- | --- | --- |
| **Biochemical parameters (mg/dL) Lanette** | | **Insulin 0,5 U/g** | **Lupeol 0,2%** | **Sham** |
| AST | 286,30 ± 35,54* | 275,90 ± 33,23* | 317,60 ± 48,19* | 151,0 ± 17,76 |
| ALT | 1153 ± 261,61*** | 717,30 ± 103.90* | 738,60 ± 184,50* | 88,50 ± 17,52 |
| γ-GT | 13,71 ± 3,41* | 11,13 ± 2,47* | 10,86 ± 3,08* | 1,42 ± 0,42 |
| Alkaline phosphatase | 761,42 ± 75,90*** | 807,61 ± 55,74*** | 687,64 ± 137,10*** | 78,86 ± 7,52 |
| Creatinine | 0,39 ± 0,03 | 0,39 ± 0,01 | 0,45 ± 0,01 | 0,40 ± 0,02 |
| Urea | 74,91 ± 8,76*** | 69,83 ± 3,46*** | 74,76 ± 4,41*** | 37,63 ± 0,91 |

**Table S2:** Biochemical parameters of rats treated with lanette, insulin 0.5 U/g or lupeol 0.2% for 14 days of experimentation (n=8). **p* <0.05 and ****p* <0.001 vs. sham group, using ANOVA followed by the Newman-Keuls test.

| **Groups** | **Day 3**  (post wounding)  **Water intake (mL)** | **Day 5** (post wounding)  **Water intake (mL)** | **Day 7**  (post wounding)  **Water intake (mL)** | | **Day 9**  (post wounding)  **Water intake (mL)** | **Day 11**  (post wounding)  **Water intake (mL)** | **Day 13**  (post wounding)  **Water intake (mL)** |  |
| --- | --- | --- | --- | --- | --- | --- | --- | --- |
| **Lanette** | 280,0 ± 33,33*** | 301,3 ± 40,84*** | 331,9 ± 46,69*** | 444,3 ± 29,26*** | | 442,3 ± 24,90*** | 477,0 ± 21,53*** |  |
| **Insulin 0.5 U/g** | 396,30 ± 26,10*** | 413,1 ± 13,29*** | 423,8 ± 10,25*** | 521,3 ± 12,02*** | | 486,9 ± 14,27*** | 527,8 ± 14,07*** |  |
| **Lupeol 0.2%** | 413,11 ± 26,59*** | 425,0 ± 16,31*** | 400,6 ± 35,70*** | 509,3 ± 17,02*** | | 458,6 ± 25,53*** | 458,8 ± 66,52*** |  |
| **Sham** | 114,30 ± 9,02 | 64,29 ± 3,68 | 82,14 ± 9,24 | 113,6 ± 9,55 | | 80,00 ± 6,075 | 106,6 ± 8,70 |  |

**Table S3:** Water intake (mL) in hyperglycemic rats treated with lanette, insulin 0.5 U/g or lupeol 0.2% during 14 days of experimentation (n=8). ****p* <0.001 vs. sham group, using ANOVA followed by the Newman-Keuls test.

| **Groups** | **Day 3**  (post wounding)  **Food intake (g)** | **Day 7** (post wounding)  **Food intake (g)** | **Day 10**  (post wounding)  **Food intake (g)** | **Day 13**  (post wounding)  **Food intake (g)** |  |
| --- | --- | --- | --- | --- | --- |
| **Lanette** | 46,25 ± 5,756*** | 135,8 ± 9,874** | 185,6 ± 8,594** | 150,3 ± 13,97** |  |
| **Insulin 0.5 U/g** | 61,75 ± 61,75*** | 144,1 ± 11,37** | 173,1 ± 13,36** | 177,6 ± 10,41* |  |
| **Lupeol 0.2%** | 69,00 ± 6,58*** | 149,5 ± 10,72* | 171,3 ± 14,30** | 142,6 ± 24,48** |  |
| **Sham** | 144,6 ± 8,92 | 215,4 ± 22,48 | 249,0 ± 14,74 | 251,9 ± 26,12 |  |

**Table S4:** Food intake (g) in hyperglycemic rats treated with lanette, insulin 0.5 U/g or lupeol 0.2% during 14 days of experimentation (n=8). **p* <0.05, ***p* <0.01 and ****p* <0.001 vs. sham group, using ANOVA followed by the Newman-Keuls test.
